# A community based field research project investigating anaemia amongst young children living in rural Karnataka, India: a cross sectional study

**DOI:** 10.1186/1471-2458-9-59

**Published:** 2009-02-17

**Authors:** Sant-Rayn Pasricha, Varalaxmi Vijaykumar, NS Prashanth, H Sudarshan, Beverley-Ann Biggs, Jim Black, Arun Shet

**Affiliations:** 1 The Nossal Institute for Global Health, Faculty of Medicine, Dentistry and Health Sciences, The University of Melbourne, Carlton, Victoria 3010, Australia; 2 The Karuna Trust, B.R.Hills, Chamarajanagar, Karnataka, India; 3 Department of Medicine (RMH/WH), Royal Melbourne Hospital, Parkville, Victoria 3050, Australia; 4 St Johns Research Institute, Sarjapur Road, Bangalore, Karnataka 560034, India

## Abstract

**Background:**

Anaemia is an important problem amongst young children living in rural India. However, there has not previously been a detailed study of the biological aetiology of this anaemia, exploring the relative contributions of iron, vitamin B12, folate and Vitamin A deficiency, inflammation, genetic haemoglobinopathy, hookworm and malaria. Nor have studies related these aetiologic biological factors to household food security, standard of living and child feeding practices. Barriers to conducting such work have included perceived reluctance of village communities to permit their children to undergo venipuncture, and logistical issues. We have successfully completed a community based, cross sectional field study exploring in detail the causes of anaemia amongst young children in a rural setting.

**Methods and design:**

A cross sectional, community based study. We engaged in extensive community consultation and tailored our study design to the outcomes of these discussions. We utilised local women as field workers, harnessing the capacity of local health workers to assist with the study. We adopted a programmatic approach with a census rather than random sampling strategy in the village, incorporating appropriate case management for children identified to have anaemia. We developed a questionnaire based on existing standard measurement tools for standard of living, food security and nutrition. Specimen processing was conducted at the Primary Health Centre laboratory prior to transport to an urban research laboratory.

**Discussion:**

Adopting this study design, we have recruited 415 of 470 potentially eligible children who were living in the selected villages. We achieved support from the community and cooperation of local health workers. Our results will improve the understanding into anaemia amongst young children in rural India. However, many further studies are required to understand the health problems of the population of rural India, and our study design and technique provide a useful demonstration of a successful strategy.

## Background

Anaemia is highly prevalent in India. The third National Family Health Study (NFHS-3) conducted during 2005–6 found that amongst children aged 6 to 59 months, the prevalence of anaemia is 69.5%; in rural India, the prevalence is 71.5%. The prevalence is maximal amongst younger children (12–17 months – 84.5%, 18–23 months 81.6%). The prevalence of anaemia in rural areas appeared to have risen since the previous NFHS (in 1998–9) [1-3].

Iron deficiency has been considered the dominant contributor to this burden. While studies conducted in urban slums have found that iron deficiency is a major cause [4], Vitamin B12 and folate deficiency have also been identified as important [5,6]. Internationally, malaria, vitamin A deficiency and genetic haemoglobinopathies significantly contribute to anaemia amongst young children [7,8]. Understanding the contribution of these causes to the overall burden of anaemia is crucial to the development of an appropriate anaemia control strategy – unlike for pregnant women, anaemia control for young children is not a cornerstone of the Reproductive and Child Health programme.

However, studies utilising modern laboratory techniques exploring the contributions of these factors to the burden of anaemia in young children of rural India are not presently available in the indexed literature [9]. Such a study is necessary in India as anaemia is most prevalent in rural children aged 12 to 23 months, and the majority of India's population is rural. There may be significant differences between rural and urban settings. A rural study may not have been conducted due to perceived logistic or community barriers. In particular, there have been concerns regarding community and family acceptability of venipuncture from young children in rural settings. Other concerns include processing and timely transport of specimens, maintenance of adequate quality control, and acceptability and tolerability of survey sampling strategies.

We have conducted a community based, cross sectional study designed to comprehensively evaluate the causes of anaemia for young children aged 12 to 23 months living in two districts of rural Karnataka, India. In this paper, we describe our detailed objectives, scientific methods, and also our processes of community consultation during the development and conduct of the study.

## Methods and design

### Study objectives

**Figure 1:**
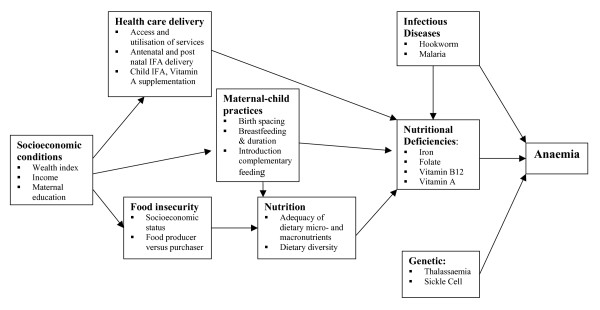
**Diagram depicting hypothesised associations of the anaemia**.

Our primary objective was to identify the prevalence of iron, vitamin B12, folate and vitamin A deficiencies, genetic haemoglobinopathies, malaria and hookworm in a population of 12–23 month old children living in rural Karnataka, and to determine the contribution of these to the overall burden of anaemia and haemoglobin levels. Finally, we sought to establish whether there were associations between these conditions and more distal risk factors, such as diet, food insecurity, standard of living, health services, and maternal nutritional status and maternal anaemia. These potential associations are depicted in Figure 1. We also intended to evaluate the contribution of these risk factors to adverse growth effects in terms of stunting, underweight and wasting. Of particular interest were associations between child feeding practices and breast feeding practices and outcomes such as anaemia, micronutrient deficiency and growth. We also sought to evaluate the delivery of current anaemia prophylaxis/therapy strategies.

### Setting

The study was based in two rural Primary Health Centres (PHCs) in southern Karnataka. The PHC is the basic functional unit for primary health within the Indian health system, each providing medical care for a defined group of villages usually comprising 20,000 to 30,000 people and serving as a hub for 4 to 6 sub-centres staffed by a female and male health worker, from which daily field operations are based [10].

The Gumballi PHC, in Chamarajnagar district, 180 km south of Bangalore, provides care for 21,700 people in 13 villages. The Sugganahalli PHC is 90 km northwest of Bangalore in Ramnagara district, and provides care for 14,400 people in approximately 80 villages [11]. Agriculture comprises the major economic activity in both regions. The average rural income in Chamarajnagar in 2006 was (Indian rupees) Rs 22,006 per capita, in Ramnagara was Rs 26,009 [12], compared with Karnataka overall Rs 26,123 [13]. These PHCs and districts were selected due to their representativeness of their state as a whole, the support for the study by local health workers, and their accessibility to Bangalore. The PHCs are managed in accordance with standard government procedures by a Non-Governmental Organization, The Karuna Trust, which has established a public-private partnership to run Primary Health Centres for the Government of Karnataka.

### Community Engagement

Participation by local families would require optimal community engagement [14]. Initial meetings were conducted to discuss the project with health and Integrated Child Development Scheme (Anganwadi) workers. The field team made two visits to each village. During the first visit, the team met with the Sarpanch (village head), the Gram Panchayat (village leadership group), the woman's sangha (female leadership committee), and other community members. Posters explaining the project were placed at Anganwadi centres. A list of children in the village was prepared. On the second visit, the team walked through the village, distributing flyers, and spoke to members of the community, especially mothers of children.

Based on these discussions, we realised that random sampling for children would be unsuccessful, as it would not be possible to gain cooperation of local leaders, parents and elders for random selection of some children but not others. A census approach, recruiting all children within a defined area, was favoured. The volume of blood to be collected was discussed with local medical staff, health workers, and villagers. It was decided that 3 mL, less than the volume of a teaspoon, would be the maximal acceptable blood draw from a young child, and would be sufficient for the laboratory for the assays.

### Field Team

The field workers were women from the local district, and each had a background in community work or health care. A female laboratory technician, again who grew up in a village, with extensive experience in paediatric venipuncture, was selected. The team worked in close collaboration with PHC staff – the Auxiliary Nurse Midwife and the Male Health Worker, as well as the Anganwadi workers, all of whom were familiar with the mothers and children in each village and could provide local guidance.

### Study Design

**Figure 2:**
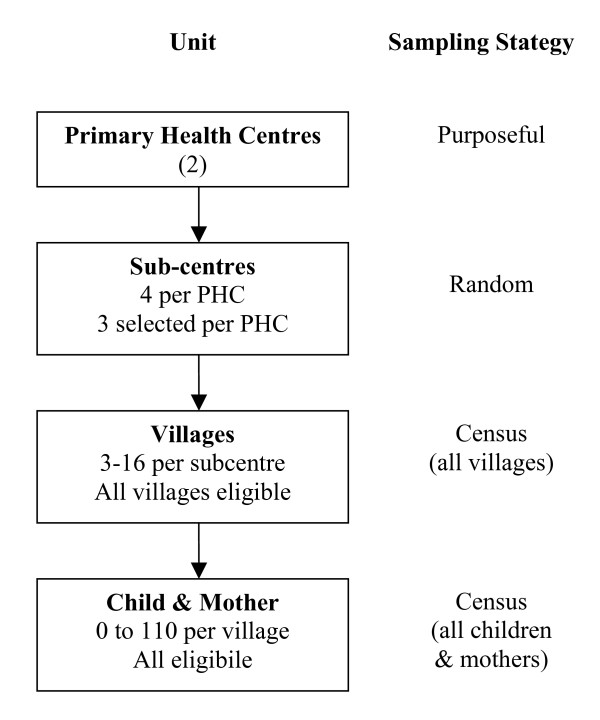
**Sampling strategy**.

A cross sectional study, aiming to recruit all children aged 12 to 23 months living in villages of selected sub-centres. From each PHC, three sub-centres were selected using a random sequence generator [15] (figure 2).

### Study Participants

Children were eligible if the village was their mother or father's home and they had slept there the previous night. Lists of children were developed from health worker and Anganwadi worker lists, as well as house to house surveys. Where exact birth dates were unavailable, ages were estimated using a local festival calendar. Children were excluded if they were found to have fever, tachypnoea, diarrhoea, clinical dehydration, or any other acute illness; or if they had previously ever received a blood transfusion.

### Questionnaire

The questionnaire (Additional File) covered basic demographic information, antenatal care, breast and complementary feeding, and interactions with the health system. The questionnaire also included modules to evaluate food security, diet, and standard of living.

The food security module was adapted from the Household Food Insecurity Access Scale, developed by the Food and Nutrition Technical Assistance project. The scale covers three domains of food insecurity: anxiety and perceptions about food insecurity, insufficient quality of food, and insufficient food intake, and enables calculation of a score [16]. The scale has been validated in previous field studies [17].

The 24 hour food recall is a simple tool for dietary assessment. Interviewees were asked to recall the exact contents of their child's diet over the previous 24 hours, using standardised cups, bowls and spoons for estimation of food volume. Nutrient content of these foods were estimated by observing local villagers prepare commonly consumed dishes, with each ingredient weighed and measured. The nutritional content of each dish was then calculated using standardised tables [18]. The 24 hour food recall has been validated against 'gold standard' strategies for dietary analysis and although found to generally overestimate the absolute nutrient content of the diet, gives results which correlate with the gold standard [19-21].

The standard of living index was adapted from the tool used for the NFHS-3. This index adopts an asset based estimation of household wealth. Interviewees are asked about dwelling characteristics and ownership of a range of household goods. Each household asset is assigned a weighting [22]. Asset ownership has been associated with consumption and with health outcomes and are more reliable in developing settings than measures of income or consumption [23].

The questionnaire was developed, piloted in the villages, revised, translated into Kannada (the local language) and then reverse translated into English to ensure accuracy.

### Anthropometry

Measurements of child length (crown-heel) were made using a portable infantometer (Seca 210, Hamburg, Germany), mother's height with a portable stadiometer (Seca 214, Hamburg, Germany), and mother and child's weight with a mother-infant scale (Seca 872, Hamburg, Germany). Growth (height-for-age, weight-for-age, and weight-for-length) was evaluated using the World Health Organization Child Growth Standards 2006 [24].

### Laboratory Processes – Preanalytical

From the mother, capillary blood was collected by fingerprick from the lateral aspect of the third finger, applied to a cuvette, and measured using the HemoCue (HemoCue 201+, Angelholm, Sweden). This technique uses the azidmethemoglobin technique for measuring haemoglobin, and is well established for field studies [25].

From the child, 3 mL of venous blood was collected from the cubital fossa or dorsum of the hand using needle/syringe or butterfly/syringe. Blood was allocated as follows: K2 EDTA microtainer (0.5 mL), serum tube with gel clot activator (2.5 mL), malaria slide (thick and thin films), and HemoCue cuvette (any residual blood).

Storage duration and conditions may influence the haematological and biochemical variables that were under study [26]. Evidence suggests that haemoglobin, red cell count and mean cell haemoglobin are stable for up to one week [27], but mean cell volume (MCV), platelet and white cell counts (WBC) alter after about 24 hours storage [28]. Serum ferritin remains stable for at least two weeks at room temperature and especially refrigerated (4°C) or frozen (-6°C) [29]. Some studies have found that serum B12 and folate are highly unstable, and that specimens must be protected from light if not assayed within 24 hours [30], although others have found that ferritin and B12 are stable in serum stored for 11°C for 14 days [31]. It was for this reason that we did not utilize the MCV, WBC and platelet counts and elected to assay our haematological and biochemistry samples daily rather than in a single batch. Retinol Binding Protein (RBP) has been shown to be an excellent and stable surrogate for retinol in assessment of Vitamin A status [32].

Samples were transported to the PHC within 4 hours of collection. EDTA tubes were refrigerated at 4°C. Serum tubes were centrifuged at 2000 rpm for 10 minutes. The serum was decanted into two microcentrifuge tubes (A and B specimens), and the A tube protected from light with aluminium foil. Specimens were then transported the following morning in an insulated ice box. This strategy was evaluated prior to commencement of the study by comparing trial samples drawn from the same subject, one processed immediately, a second drawn in the field and processed using the above protocol. There was no deterioration in haemoglobin, ferritin, serum B12, folate or C-reactive protein,

### Laboratory Processes – Analytical

The following assays were performed: full blood examination (Sysmex XT-2000i, Sysmex Inc., Kobe, Japan); serum ferritin, serum folate and serum B12 (electrochemiluminescent immunoassay, ELECSYS 2010, Hitachi High Technologies Corporation, Tokyo, Japan; reagents from Roche Diagnostics, Penzberg, Germany), retinol binding protein and high sensitivity C-reactive protein (nephelometry, Siemens BN-Prospec Nephelometer, Marburg, Germany), haemoglobin variant (high performance liquid chromatography, Biorad D10, Biorad Laboratories Inc., Hercules, USA). Thick and thin malaria slides were stained (Jaswant Singh Battacharya (JSB) stain) and evaluated in the field by National Malaria Control Programme accredited microscopists.

Mothers were advised to collect the stool on a square of newspaper and transfer it to the container using a wooden paddle, which was provided. Stool specimens were preserved in 10% formalin in saline within six hours of collection. Stools were examined for parasites by wet mount with normal saline [33].

### Field Processes

The field work in each village was conducted across three days. On the first afternoon, the team visited the village, and met local women and children, guided by the local health worker or Anganwadi worker. The team explained the study, the rationale, risks and potential benefits. The following morning, mothers were registered and interviews conducted. Following the questionnaire, the child and mother were taken to the Anganwadi centre, where anthropometric measurements were conducted and blood collected in privacy. On the third day, a team member returned to the village to retrieve collected stools.

### Quality Control

Measures were adopted to achieve optimal quality of questionnaire, anthropometry and laboratory data. Following recruitment, the field team received extensive training in the theme and objectives of the study and the concept of ethical research. Administration of the questionnaire, especially the 24 hour food recall and food security modules, was taught, Each interviewer practiced the questionnaire with the field coordinator and then administered the questionnaire with a trial subject from a non-selected village [34]. All completed questionnaires were reviewed daily for mistakes and anomalies. Finally, cross checks were conducted to compare the results of questionnaires between interviewers on the same subjects. Anthropometry was performed by the laboratory technicians trained in the performance of these measurements. The apparatus were new and calibrated by the manufacturer prior to delivery. At least 10% of anthropometric measurements were double checked by the researcher.

All laboratory specimens were run approximately 30 hours following blood collection. Laboratory instruments were calibrated daily using standards provided by the manufacturer. Three controls (low, normal and high ranges) were run for ferritin, serum B12, folate, and retinol binding protein. Approximately 30% of stool specimens were examined independently by two staff medical microbiologists.

### Ethics

Ethics approval was received from the Institutional Ethics Review Board of St John's National Academy of Health Sciences and the Human Research Ethics Committee of the University of Melbourne. The project was also approved by the board of the Karuna Trust. Written informed consent (translated into the local language) was obtained from all mothers and plain language statements distributed.

An incentive comprising a small toy, packet of biscuits and a bedspread, not exceeding INR100 (US$2.00) was provided to each family. Paediatric iron drops were supplied to anaemic children, distributed by local health (not study) workers. All participants received a written copy of their blood results.

### Statistical analysis

Assuming a birth rate of 20/1000, approximately 700 eligible children live in the area (total population 35,000). Assuming a prevalence of proportions of interest of 50%, a sample size of 390 would enable a confidence interval +/-5% for estimates of prevalence. By selecting 6 of 8 sub-centres we anticipated 525 children would be eligible. A non response rate of 20% would result in a sample of 420 children, achieving our target sample size. This sample size would also enable an estimate of associations between independent and dependent variables where the odds ratio is at least 1.8 and the ratio of cases to controls of 3:7 (based on an expected prevalence of anaemia of 70–80%); and an estimate of regression equations between continuous variables where the slope is 0.1 and correlation between dependent and independent variables 0.2; with a power of 0.8.

All variables were examined for normality and logarithmically transformed where appropriate. Descriptive parametric statistics were calculated. Associations between continuous variables were estimated using linear regression and binary variables using logistic regression. T-tests were used to identify differences in continuous variables between binary groups.

## Results

**Figure 3:**
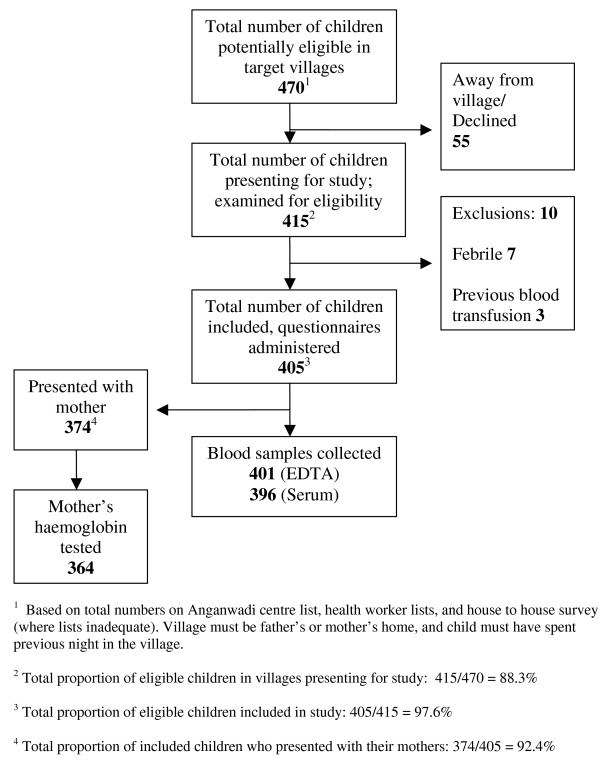
**Diagram of recruitment of study subjects**.

Figure 3 depicts the recruitment. Over 88% of the eligible children identified to be residing in the villages were recruited for the study. Questionnaire data was obtained from 405 children, venous blood for haemoglobin from 401, and serum from 396.

## Discussion

We have conducted a study exploring anaemia amongst young (12–23 month) children living in two districts of rural Karnataka, the first such study conducted in rural India.

Although random sampling would have enabled us to provide estimates across a larger area and sample space, we found it difficult to explain the concept of random sampling in the villages. Selecting some but not all children in a village may have generated suspicion. Selecting villages randomly as clusters may have had a similar effect as subcentre workers would have been unable to conceal activities occurring in one village from the neighbouring communities. This may have resulted in repercussions between the community and health workers. By selecting at the level of the sub-centres, each with their independent infrastructure and personnel, the study could adopt a 'programme' approach, and recruit participants, collect data and advise treatment accordingly. Providing treatment for anaemic children across the villages ensured there was reciprocal health benefit to participants.

Employing women drawn from the villages and gaining assistance from local health workers may have improved the acceptability of the study to local mothers. Providing training, ensuring rehearsal, maintaining daily review of questionnaires, and providing frequent feedback ensured adequate data quality was maintained.

Although we had a good response in terms of questionnaires and blood collection, fewer than half the participants returned stool samples. This appeared to be due to distaste for collecting the samples, and secondly, forgetting or lacking the time to return the samples to the study team at the time requested.

## Conclusion

In conclusion, we have conducted a community based study to understand anaemia amongst rural children. India is a large and diverse country, with a high burden of anaemia. It is unlikely our study is generalizable to all regions, and thus there is a need for further studies in rural India. Our methodology and field strategies could serve as a template for epidemiologic, clinical and policy related research into anaemia and other health problems in young rural children.

## Competing interests

The authors declare that they have no competing interests.

## Authors' contributions

SP, HS, JB, BB and AS conceived and designed the study. SP and VV developed the field methodology and directed the field work. SP, NP, JB, BB and AS evaluated the data and conducted quality control. All authors read and approved the final manuscript.

## Pre-publication history

The pre-publication history for this paper can be accessed here:



## Supplementary Material

Additional file 1 **Questionnaire.** The questionnaire measured demographics, standard of living. Click here for file

## References

[B1] IIPS (2007). National Family Health Survey (NFHS-3), 2005–06.

[B2] (2005). Fact Sheet Karnataka. National Family Health Survey (NFHS-3).

[B3] IIPS (2000). National Family Health Survey (NFHS-2) 1998–99.

[B4] Kapur D, Agarwal KN, Sharma S, Kela K, Kaur I (2002). Iron status of children aged 9–36 months in an urban slum Integrated Child Development Services Project in Delhi. Indian Pediatr.

[B5] Gomber S, Kumar S, Rusia U, Gupta P, Agarwal KN, Sharma S (1998). Prevalence & etiology of nutritional anaemias in early childhood in an urban slum. Indian J Med Res.

[B6] Taneja S, Bhandari N, Strand TA, Sommerfelt H, Refsum H, Ueland PM, Schneede J, Bahl R, Bhan MK (2007). Cobalamin and folate status in infants and young children in a low-to-middle income community in India. Am J Clin Nutr.

[B7] Calis JCJ, Phiri KS, Faragher EB, Brabin BJ, Bates I, Cuevas LE, de Haan RJ, Phiri AI, Malange P, Khoka M (2008). Severe Anemia in Malawian Children. New England Journal of Medicine.

[B8] Thurlow R, Winichagoon P, Green T, Wasantwisut E, Pongcharoen T, Bailey KB, Gibson RS (2005). Only a small proportion of anemia in northeast Thai schoolchildren is associated with iron deficiency. Am J Clin Nutr.

[B9] Singh P, Toteja GS (2003). Micronutrient profile of Indian children and women: summary of available data for iron and vitamin A. Indian Pediatr.

[B10] Primary Health Care Resources in India. http://www.whoindia.org/LinkFiles/Health_Systems_Development_Primary_Health_Care_Primary_health_care_resources_.pdf.

[B11] (2008). PHC Monthly Data – November 2007.

[B12] Universal Currency Calculater. http://xe.com.

[B13] Bhandari L, Kale S (2007). Karnataka: Performance, Facts and Figures.

[B14] Macaulay AC, Commanda LE, Freeman WL, Gibson N, McCabe ML, Robbins CM, Twohig PL (1999). Participatory research maximises community and lay involvement. BMJ.

[B15] random.org. http://www.random.org/sequences/.

[B16] Coates J, Swindale A, Bilinsky P (2007). Household Food Insecurity Access Scale (HFIAS) for Measurement of Food Access: Indicator Guide. Academy for Educational Development.

[B17] Swindale A, Bilinsky P (2006). Development of a Universally Applicable Household Food Insecurity Measurement Tool: Process, Current Status, and Outstanding Issues. J Nutr.

[B18] Gopalan C, Sastri BR, Balasubramanian S (1971). Nutritive Value of Indian Foods.

[B19] Horst CH, Obermann-De Boer GL, Kromhout D (1988). Validity of the 24-Hour Recall Method in Infancy: The Leiden Pre-School Children Study. International Journal of Epidemiology.

[B20] Fisher JO, Butte NF, Mendoza PM, Wilson TA, Hodges EA, Reidy KC, Deming D (2008). Overestimation of infant and toddler energy intake by 24-h recall compared with weighed food records. American Journal of Clinical Nutrition.

[B21] Olinto MTA, Victora CG, Barros FC, Gigante DP (1995). Twenty-Four-Hour Recall Overestimates the Dietary Intake of Malnourished Children. Journal of Nutrition.

[B22] Rutstein SO, Johnson K (2004). The DHS Wealth Index. DHS Comparative Reports No 6.

[B23] Montgomery MR, Michele Gragnolati, Burke KA, Paredes E (2000). Measuring Living Standards with Proxy Variables. Demography.

[B24] (2006). The WHO Child Growth Standards.

[B25] (2004). Assessing the iron status of populations: Report of a Joint World Health Organization/Centers for Disease Control and Prevention Technical Consultation on the Assessment of Iron status at the population level.

[B26] Narayanan S (2000). The Preanalytic Phase: An Important Component of Laboratory Medicine. Am J Clin Pathol.

[B27] Gulati GL, Hyland LJ, Kocher W, Schwarting R (2004). Changes in Automated Complete Blood Cell Count and Differential Leukocyte Count Results Induced by Storage of Blood at Room Temperature. Arch Pathol Lab Med.

[B28] Buttarello M (2004). Quality specification in haematology: the automated blood cell count. Clinica Chimica Acta.

[B29] Kubasik NP, Ricotta M, Hunter T, Sine HE (1982). Effect of Duration and Temperature of Storage on Serum Analyte Stability: Examination of 14 Selected Radioimmunoassay Procedures. Clin Chem.

[B30] Komaromy-Hiller G, Nuttall KL, Ashwood ER (1997). Effect of storage on serum vitamin B12 and folate stability. Ann Clin Lab Sci.

[B31] Drammeh BakaryS, Schleicher RosemaryL, Pfeiffer ChristineM, Jain RamB, Zhang Mindy, Hong Phuong Nguyen (2008). Effects of Delayed Sample Processing and Freezing on Serum Concentrations of Selected Nutritional Indicators. Clin Chem.

[B32] Gamble MV, Ramakrishnan R, Palafox NA, Briand K, Berglund L, Blaner WS (2001). Retinol binding protein as a surrogate measure for serum retinol: studies in vitamin A-deficient children from the Republic of the Marshall Islands. American Journal of Clinical Nutrition.

[B33] Ash LR, Orihel TC, Savioli L, World Health O (1994). Bench Aids for the Diagnosis of Intestinal Parasites.

[B34] McBurney DH, White TL (2007). Research Methods.

