# Supplementary material for: A community based field research project investigating anaemia amongst young children living in rural Karnataka, India: a cross sectional study
Source: BMC Public Health. 2009 Feb 17;9:59. doi: 10.1186/1471-2458-9-59 (PMC2667415; doi:10.1186/1471-2458-9-59)
Supplement: Additional file 1 — Questionnaire. The questionnaire measured demographics, standard of living. [file 1471-2458-9-59-S1.doc]

**CHILD’S NAME: ID Number:**

**MOTHER/ CARER’S NAME:**

|  |  | **Code** |
| --- | --- | --- |
| District | Gumballi  Sugganahalli |  |
| Village | **Village Name**  **Anganwadi Centre** |  |
| Date of Interview: |  |  |
| Time: |  |  |
| Interviewer: |  |  |
| Completed: | Y / N |  |
| Data entry | A SRP  B Other _____________ |  |

1. Inclusion and Exclusion criteria

|  | Inclusion Criteria | Options | Response | Code |
| --- | --- | --- | --- | --- |
| A1a | Child’s date of birth | ___/___/___  Unsure (Ask A1b) | If not 12-24 months of age, exclude. |  |
| A1b | Was child born between  _____/06 and ____/07 | Yes  N (**exclude**)  Unsure (Ask A1c) |  |  |
| A1c | Was the child born after  _______________ (local calendar). | Y  N -> **Exclude**  Unsure -> **Exclude!** |  |  |
| A2 | Child’s age in months | ___________ months |  |  |
| A3 | Is this your child? | Y  N -> Don’t ask Section C questions |  |  |

|  | **Exclusion Criteria**  **(with doctor)** | **Options** | **Response** | **Code** |
| --- | --- | --- | --- | --- |
| *A4 | Does the child have fever now? | Y -> exclude  N |  |  |
| A5 | Does the child have fast breathing today? | Y -> exclude  N |  |  |
| *A6 | Does the child have diarrhoea (>6 stools per day) now? | Y -> exclude  N |  |  |
| A7 | Dehydration (moderate or severe) now? | Y -> exclude  N |  |  |
| A8 | Drowsiness/ fatigue/ lethargy | Y -> exclude  N |  |  |
| *A9 | Has the child ever had a previous blood transfusion? | Y -> exclude  N |  |  |

**B. Socio-**demographic Questions (Mother)

|  |  | Options | Response | Code |
| --- | --- | --- | --- | --- |
| B1 | How old are you (mother)? | Age ___________ |  |  |
| B2 | What is your caste? | Scheduled Caste  Scheduled Tribe  Other (Non SC/ T) |  |  |
| B3 | How many years of school did you finish? | ___________years |  |  |
| B4 | Can you read and write? | Yes  No |  |  |

**C. Anaemia risk factors (mother)**

These questions are related to things which can strengthen or weaken your blood.

|  |  | Options | Response | Code |
| --- | --- | --- | --- | --- |
| C1 | Could you tell me the birth order of **this** child?  (Please include all children you have given birth to.) | Number: _________ |  |  |
| C2 | How many pregnancies have you had so far? | Number __________ |  |  |
| C3 | At the moment, are you pregnant? | Y  N |  |  |
| The next questions are about when you were pregnant with **this** (Name _________________) child. | | | | |
| C4 | What is the age gap between this child and the previous child? | __________ Months  Or   Oldest child |  |  |
| C5 | Whilst you were pregnant with **this** child, how many times did you see any midwife, doctor or health worker? | __________ Times |  |  |

| C6 | During your pregnancy with this child, do you recall being given iron/ folic acid tablets by any person? (Visual Cue) | Y  N (-> go to C9) |  |  |
| --- | --- | --- | --- | --- |
| C7 | If yes, approximately how many iron/ folic acid tablets were you given during that pregnancy (total)?  (visual cue) – number of small strips (each strip = 10 tablets) | A. 10-20 (1-2 strips)  B. 30-40 (3-4 strips)  C. 50-60 (5-6 strips)  D. 70-80 (7-8 strips)  E. >80 (>8 strips) |  |  |
| C8 | If yes – how many tablets did you actually take during that pregnancy? | A. 10-20 (1-2 strips)  B. 30-40 (3-4 strips)  C. 50-60 (5-6 strips)  D. 70-80 (7-8 strips)  E. >80 (>8 strips) |  |  |
| C9 | During your last pregnancy, do you recall having a blood test early/ when the pregnancy was detected? | Y  N  Can’t Remember |  |  |

| C10 | Since the end of your last pregnancy, have you had any blood test? | Y  N  Can’t Remember |  |  |
| --- | --- | --- | --- | --- |
| C11 | Since your last pregnancy, have you seen any doctor/ midwife/ health worker about **your** health? | Y  N |  |  |
| C12 | Since your last pregnancy, have you taken any iron/ folic acid tablets? | Y  N |  |  |
| C13 | If yes, approximately how many strips have you taken? | A. 10-20 (1-2 strips)  B. 30-40 (3-4 strips)  C. 50-60 (5-6 strips)  D. 70-80 (7-8 strips)  E. >80 (>8 strips) |  |  |

1. Anaemia risk factors (child)

|  |  | Options | Response | Code |
| --- | --- | --- | --- | --- |
| D1 | Child’s sex | Male  Female |  |  |
| D2 | Did you breast feed this child? | Y  N |  |  |
| D3 | For how many months did you give the child breast milk alone, with no other foods (exclusive breastfeeding)? | Months _________  (round to nearest) |  |  |
| D4 | Are you still breastfeeding this child? | Y -> to D6  N |  |  |
| D5 | At what age did you stop breastfeeding altogether? | Age ______ (months) |  |  |
| D6 | Have you yet introduced other foods for the child? | Y  N -> D8 |  |  |
| D7 | At what age did you first give other foods for this child? | Age _______ (months) |  |  |

| D8 | Has your child ever been seen by a doctor? | Y  N  Unsure |  |  |
| --- | --- | --- | --- | --- |
| D9 | Has your child ever been to the PHC? | Y  N  Unsure |  |  |
| D10 | Has your child ever been to the Anganwadi centre? | Y  N  Unsure |  |  |
| D11 | Has the child ever had a blood test before? | Y  N  Unsure |  |  |
| D12 | Has the child ever received iron/ folic acid tablets or syrup  (visual cue) | Y  N  Unsure |  |  |

| D13 | If yes, from whom? | 1. Auxiliary Nurse Midwife 2. Anganwadi worker 3. Health worker at Sub centre 4. PHC 5. From private shop, non health worker initiated 6. Private doctor 7. Can’t remember |  |  |  |
| --- | --- | --- | --- | --- | --- |
| D14 | If tablets, how many tablets were given? | A. <10   1. 10-20 (1-2 strips)   C. 30-40 (3-4 strips)  D. 50-60 (5-6 strips)  E. 70-80 (7-8 strips)  F. >80 (>8 strips) |  |  |  |
| D15 | If tablets were given, how many did your child actually take? | A. <10   1. 10-20 (1-2 strips)   C. 30-40 (3-4 strips)  D. 50-60 (5-6 strips)  E. 70-80 (7-8 strips)  F. >80 (>8 strips) |  |  |  |
| D16 | Has your child ever received Vitamin A liquid or capsules (visual cue) | Y  N  Unsure |  |  |  |
| D17 | If yes, how many times?  (should be 1 -3) | _________ |  |  |  |
| D18 | Which vaccinations has your child received (tick those which have been received): | 0 (birth)   6 weeks (1.5 mo)   10 weeks (2.5 mo)   14 weeks (3.5 mo)   9 months   16-18 months  |  |  |  |
| D19 | Is your child walking? | Y:    N: |  |  |  |
| D20 | In the past month, how many take home rations have you received from the Anganwadi centre? | __________ (number) |  |  |  |

1. **Food Security**

For the following questions, we are asking about your household: anyone who sleeps in your house and who shares food in the family regularly. Please be as truthful as you can – we will keep the answers secret.

|  |  | Options | Response | Code |
| --- | --- | --- | --- | --- |
| E1 | In the past four weeks, did you worry that your home would not have enough food?  (If yes, then how often in one month?) | 1. No 2. 1 or 2 times in the past month 3. three to ten times in the past month 4. more than ten times in the past month |  |  |
| E2 | In the past four weeks, was here a time when you or someone in the house could not eat what food they wanted because of not enough resources/ money?  (If yes, then how often in one month?) | 1. No 2. 1 or 2 times in the past month 3. three to ten times in the past month 4. more than ten times in the past month |  |  |

| E3 | In the past four weeks, did you or anyone in the house have no choices or restrict the variety of the food you ate because of lack of resources/ money?  (If yes, then how often in one month?) | 1. No 2. 1 or 2 times in the past month 3. three to ten times in the past month 4. more than ten times in the past month |  |  |
| --- | --- | --- | --- | --- |
| E4 | In the past four weeks, did you or someone in the house have to eat some food you did not like or want to eat because of lack of resources/ money to obtain other food?  (If yes, then how often in one month?) | 1. No 2. 1 or 2 times in the past month 3. three to ten times in the past month 4. more than ten times in the past month |  |  |

E5 and E6 Stem:

In the past 4 weeks, did you or someone in the house have to eat less food than you wanted because of a lack of resources:

| E5 | By having less food in a meal than they wanted?  (If yes, then how often in one month?) | 1. No 2. 1 or 2 times in the past month 3. three to ten times in the past month 4. more than ten times in the past month |  |  |
| --- | --- | --- | --- | --- |
| E6 | By having fewer number of meals in the day than you/ they wanted?  (If yes, then how often in one month?) | 1. No 2. 1 or 2 times in the past month 3. three to ten times in the past month 4. more than ten times in the past month |  |  |

| E7 | In the past four weeks, was there ever no food to eat in your house because of lack of resources/ money to get food?  (If yes, then how often in one month?) | 1. No 2. 1 or 2 times in the past month 3. three to ten times in the past month 4. more than ten times in the past month |  |  |
| --- | --- | --- | --- | --- |
| E8 | In the past four weeks, did you or anyone in the house go to sleep at night hungry because there was not enough food?  (If yes, then how often in one month?) | 1. No 2. 1 or 2 times in the past month 3. three to ten times in the past month 4. more than ten times in the past month |  |  |
| E9 | In the past four weeks, did you or anyone in the house go one whole day and night without eating anything because there was not enough food?  (If yes, then how often in one month?) | 1. No 2. 1 or 2 times in the past month 3. three to ten time in the past month 4. more than ten times in the past month |  |  |
| E10 | Do you feel that your child has enough food to eat? (quantity) | Y  N |  |  |
| E11 | Do you feel that this child has enough good, healthy (quality) food to eat? | Y  N |  |  |
| E12 | Who eats last in the house? | 1. Grandfather 2. Grandmother 3. Father 4. Mother (respondent) 5. Child other than child 6. Child selected |  |  |
| E13 | How do you usually obtain the main cereal food you eat (ragi, rice, atta) | 1. Grown by family 2. Purchased 3. Food for labour |  |  |

**F. 24 hour dietary recall (child)**

Was yesterday a typical day in terms of what your child had to eat? Y/N

If Yes -> recall yesterday.

If No: recall most recent typical day (write how many days ago: ____________)

Please try to remember exactly what your child had to eat yesterday (above date). Start from early morning, and list each item until the child went to sleep in the evening. We would like you try to estimate the quantity of each food. We have brought some utensils to help you.

Please try to remember: (add to the table)

Did your child have any breast milk (when)?

Did your child have any biscuits (brand)?

Did your child have any candies/ lollies?

Did your child have any other food or snacks?

**F1: 24 hour dietary recall OIL USED: ______________________________**

| **Time** | Food items & quantity | Remarks |
| --- | --- | --- |
| **Early morning**  **(before breakfast)** |  |  |
| **Breakfast** | How much food left uneaten? |  |
| **Morning** |  |  |
| **Lunch** | How much food left uneaten? |  |
| **Afternoon** |  |  |
| **Dinner** | How much food left uneaten? |  |
| **Evening** |  |  |
| **Extra food during day** |  |  |

Specific foods

|  |  | **Options** | **Response** | **Code** |
| --- | --- | --- | --- | --- |
| F2 | In this past one month, how many times has your child eaten:  red meat (e.g. mutton, lamb, goat, others) | _________ times |  |  |
| F3 | In this past one month, how many times has your child eaten:  white meat (e.g. chicken, fish) | __________times |  |  |
| F4 | In the past one month, how many times has your child had any meat (either red or white) | _________ time |  |  |
| F5 | In the past month, how often has your child eaten eggs (boiled, scrambled, fried, any way) | _________ times |  |  |
| F6 | In the past month, how often has your child eaten green leafy vegetables (e.g. spinach) | __________times |  |  |

|  |  | Options | Response | Code |
| --- | --- | --- | --- | --- |
| F7 | Regarding the sambar/ curry/ rasam made yesterday:  Did you use any vegetables other than onion/ ginger/ garlic? | Y  N |  |  |
| F8 | Regarding the sambar/ curry/ rasam for yesterday:  Was it thick or thin? | Thick  Thin |  |  |
| F9 | Do you sprout ragi that you give your child? | Y  N |  |  |

1. **Standard of living index**

| Number | Item | Options | Code |
| --- | --- | --- | --- |
| G1 | House type | Pucca – 4  Semi-Pucca – 2  Katcha - 0 |  |
| G2 | Is there a separate room for cooking | Yes – 1  No – 0 |  |
| G3 | How much agriculture land does this household/ family own? | 5+ Acres - 4  2-4.9 Acres -3  0.1-2 Acres – 2  No land – 0 |  |
| G4 | (Not if G3 = 0) If the family owns land, is any irrigated with water? | Some – 2  None - 0 |  |
| G5 | Does the family own the house? | Yes – 2  No – 0 |  |

| G6 | Toilet facility | Flush toilet, own – 4  Flush toilet, shared/ public ; own pit toilet -2  Shared/ public pit toilet – 1  No access to toilet/ use outside, behind a bush etc – 0 |  |
| --- | --- | --- | --- |
| G7 | Source of lighting | electricity=2  kerosene, gas, oil=1  Other source (wood, dung) – 0 |  |
| G8 | Main fuel for cooking | electricity, liquid petroleum gas or biogas=2  coal, charcoal or kerosene=1  other fuel=0 |  |

| G9 | Source of drinking water | pipe, hand pump, well in residence/ yard/ plot=2  public tap, hand pump or well=1  other water source (e.g. tanker truck, open source) =0 |  |
| --- | --- | --- | --- |
| **Do you own:** | | | |
| G10 | A tractor? | Yes- 4 |  |
| G11 | Car? | Yes – 4 |  |
| G12 | Moped or scooter? | Yes – 3 |  |
| G13 | Telephone | Yes – 3 |  |
| G14 | Refrigerator | Yes – 3 |  |
| G15 | Television | Colour - 3  Black and white – 2 |  |
| G16 | Bicycle | Yes – 2 |  |
| G17 | Electric fan | Yes – 2 |  |
| G18 | Radio | Yes – 2 |  |
| G19 | Mattress | Yes – 1 |  |
| G20 | Pressure cooker | Yes – 1 |  |
| G21 | Chair | Yes – 1 |  |
| G22 | Cot or bed | Yes – 1 |  |
| G23 | Table | Yes – 1 |  |
| G24 | Clock or watch | Yes – 1 |  |
| G25 | Livestock | Yes – 2 |  |
| G26 | Water pump | Yes – 2 |  |
| G27 | Bullock cart | Yes – 2 |  |
| G28 | Thresher | Yes – 2 |  |
| G29 | How much did you and your household earn in the last 3 months? | Rs_____________ |  |
| G30 | How do you earn your money? (choose all that apply) | Daily Wage  Monthly wage  Food for labour  Selling livestock |  |
| G31 | How many people are there who share food, eat and sleep in this home regularly every day? | Number: __________ |  |

Total SLI:

Thank you for helping us with this questionnaire. That is the end of the questions. Please now come for the measurement and blood test.

1. **Anthropometric Measurements and Laboratory Evaluation**

**Child**

| **Length (cm)** |  |
| --- | --- |
| **Weight (kg, 1 decimal place)** |  |

**Mother**

| **Height (m)** |  |
| --- | --- |
| **Weight (kg)** |  |
| **BMI (wt/ht^2^)**  **To be calculated** |  |

**Child’s venous blood sample**

| Sample collected |  | Code |
| --- | --- | --- |
| **Child** | Yes  No |  |

**Child’s Venous Haemoglobin**

**(g/dL) (HemoCue)**

**Stool Collection**

| Sample collected | Yes  No |  |
| --- | --- | --- |
| Hookworm eggs per gram | Count ____________ | Epg ______________ |
| Ascaris eggs per gram | Count ____________ | Epg ______________ |
| Trichuris eggs per gram | Count ____________ | Epg ______________ |

**Maternal Fingerprick Haemoglobin**

**(g/dL) (HemoCue)**
